# Using Data Compression to Build a Method for Statistically Verified Attribution of Literary Texts

**DOI:** 10.3390/e23101302

**Published:** 2021-10-03

**Authors:** Boris Ryabko, Nadezhda Savina

**Affiliations:** 1 Federal Research Center for Information and Computational Technologies of SB RAS, 630090 Novosibirsk, Russia; 2 Department of Information Technologies, Novosibirsk State University, 630090 Novosibirsk, Russia; nn_savina@mail.ru

**Keywords:** data compression, authorship attribution of literary texts, hypothesis testing, quantitative study of literature

## Abstract

We consider the problems of the authorship of literary texts in the framework of the quantitative study of literature. This article proposes a methodology for authorship attribution of literary texts based on the use of data compressors. Unlike other methods, the suggested one gives a possibility to make statistically verified results. This method is used to solve two problems of attribution in Russian literature.

## 1. Introduction

One of the interesting problems of quantitative linguistics and, in particular, the quantitative study of literature is the establishment of authorship of literary texts. This problem is associated with the existence of anonymous and pseudonymous texts, and is one of the oldest philological and linguistic problems. Perhaps the most famous problem of attribution of authorship is the so-called “Shakespearean question” [1]. Many researchers doubt the authorship of Shakespeare in relation to a number of works signed with his name.

To solve the problem of attribution of literary works, along with traditional literary methods, researchers began to use the approaches of such disciplines as the theory of random processes, pattern recognition and a number of other branches of science related to the artificial intelligence, see [2,3,4,5,6,7].

In this article, we develop a method for attribution of literary text based on the use of data compression techniques. The main innovation of this method is the ability to obtain statistically confirmed results based on the use of ideas and methods of information theory and mathematical statistics.

It is worth noting that the first methods of data compression (or source coding) were developed by C. Shannon in their famous article [8], and now data compressors are widely used in statistical analysis and forecasting [2], clustering [3,4,9,10,11,12,13] and some other areas far from data transfer and storage. In such applications, the text is an input for the data compressor (or archiver), which encodes this text into a file of shorter length, that is, “compresses”. This “compressed file” can be decoded into the original text by the same archiver. Compression is achieved due to the fact that archivers find the frequency of occurrence of symbols and their combinations using various methods of information theory, the theory of formal grammars and some methods of artificial intelligence.

The basic idea behind many applications of data compressors was suggested in [9,10] and can be illustrated by the following example. Suppose, there are three sequences $x=x_{1}x_{2}\ldots x_{n},y=y_{1}y_{2}\ldots y_{k},z=z_{1}z_{2}\ldots z_{m}$ and let ψ be a data compressor. It is known that the sequences x,y obey different probability distribution, while *z* obeys one of them. The goal is to determine the distribution of *z*. (It is the well-known “three-sample problem” in mathematical statistics.) The key observation is this: if *x* and *z* obey the same probability distribution, then sequence *z* will be better compressed after *x* than after *y*. That is, if |ψ(xz)|−|ψ(x)|<|ψ(yz)|−|ψ(y)|, then *x* and *z* obey the same distribution if |ψ(xz)|−|ψ(x)|>|ψ(yz)|−|ψ(y)|, then *y* and *z* obey the same distribution, where ψ(u) is the compressed sequence *u* (that is, the corresponding binary word), |vs.| is the length of vs. The point is that if *x* and *z* obey the same distribution, the second word in xz will be compressed “better” than in the word yz, because in the first case, the compression of the subword *z* is based on the true statistics (obtained by encoding the first part). For example, let *x* and *z* be English texts, and *y*—German. Then, the English text *z* will be better compressed after the text in the same language, i.e., |ψ(xz)|−|ψ(x)| will be shorter than |ψ(yz)|−|ψ(y)|.

From our point of view, it is highly desirable that mathematical approaches be developed within the framework of mathematical statistics to obtain statistically verified results. In [14], such a method was developed for a data compression approach, but it was a formal solution to the “three-sample problem” and cannot be applied as it is to the problem of authorship attribution. In this paper, we develop a compression-based “three-sample problem” method for identification of the author of literary texts. This method can be applied to literary texts in any language, but in this article we will illustrate its use for attribution problems of some famous Russian novels that arose in the 19th and 20th centuries, respectively.

In the next section, we describe two attribution issues discussed in this article. The third part contains a description of the proposed method and the solution to the first problem (we do this in parallel to make the description shorter and clearer). The fourth part contains a solution to the problem of attribution of the 19th century, and the short conclusion summarizes the main results.

## 2. Two Problems of Attribution of Literary Texts

The first problem is connected with two novels “The Twelve Chairs” and “The Golden Calf” written by I. Ilf and E. Petrov. These novels are very popular in Russia and around the world. “The Twelve Chairs” has been translated into sixty languages including English, German, Spanish, Swedish, Italian, Polish, French, Portuguese and so on. Based on the novel “The Twelve Chairs”, movies were created in 19 countries in the period from 1933 to 2013. However, suddenly, in 2013, Irina Amlinski published the book “12 chairs from Mikhail Bulgakov”. In this book, she gives arguments in favor of the fact that the books “12 Chairs” and “The Golden Calf” were not written by I. Ilf and E. Petrov, but that the real author of these novels is M. Bulgakov [15]. After that, the opinions of literary critics, writers and readers were divided. There are those who are sure that there was a hoax [16], and there are those who are sure that there was no hoax [17]. In addition, there are those who could not come to a final conclusion [18], and this discussion continues until now.

The second problem is connected with famous writers of the 19th century. Namely, the authorship attribution of N. Nekrasov and A. Panaeva (Stanitsky’s pseudonym) of several novels published in the “Sovremennik” magazine in the 19th century. For example, the authorship of the novels “Three Countries of the World” and “Dead Lake” has not been identified. Literary critic B.L.Bessonov, having carefully studied the texts of these novels, the literary work of the alleged authors, as well as their memoirs, came to the conclusion that it is impossible to fully attribute the novels to one of the authors. At the same time, the researcher made reasonable assumptions that most of the chapters of both novels belong to Nekrasov [19], but this assumption absolutely contradicts Panaeva’s “Memoirs” [20]. One of the main problems that literary critics highlight is the authorship of the first part of “Three Countries of the World”, namely the “Prologue” part. In her “Memoirs” Panaeva reports that she wrote the first part. However, the researchers came to the conclusion that the “Prologue” could not be written without a preliminary plan of the entire novel as a whole, because the “Prologue” describes events that anticipate the plot of the two penultimate parts. Therefore, the attribution of each part of the work must be carried out separately. The question of the“ shared ” participation of Panaeva and Nekrasov in the creation of works remains open at the moment. The difficulty in identifying the author of the novels is primarily due to the fact that both of them have reached us only in printed editions. No manuscripts, proofs, or other documents have been preserved, according to which it would be possible to distinguish the author’s contribution of each writer. Therefore, researchers who have addressed the problem of attribution of these novels are forced to admit that their conclusions are “very approximate”.

## 3. Description of the Method

In this part, we describe the proposed method. All steps will be illustrated with examples from the first considered problem—the attribution of the works “The Twelve Chairs” and “The Golden Calf”.

Preliminary stage. Let us give some notations. We consider situation where there are several texts written by different authors. Let $X_{i}$ be some text of the *i*-th author and let XY be the text *X*, to which the text *Y* has been assigned to the right without any additional characters. Finally, let ψ(Z) be the text *Z* compressed by a data-compressor (an archiver) ψ. The value Ψ(Y/X)=|ψ(XY)|−|ψ(X)|, called the conditional text compression ratio, characterizes the degree of closeness between *X* and *Y*. Informally, it is assumed that the better the text XY was compressed, the more information about *Y* was contained in *X*, which means that the style of this author most closely matches the style in which the text *Y* of the unknown author is written.

The preliminary stage is intended to select the parameters of the method based on experiments with the texts of writers who lived simultaneously with the investigated ones. For this purpose, we collected most of the works of Russian authors written in the period from 1915 to 1940. (The books “The Twelve Chairs” and “The Golden Calf” were published in 1927 and 1931, correspondingly). Note that poetic texts, fairy tales and children’s literature were not used. It is important to emphasize that the texts of Ilf and Petrov, as well as Bulgakov, were not included.

Then, we carried out the following preparation of the selected authors texts: we divided the texts of each author into two parts, which we called training sample and experimental one. Both parts are composed of different works by a certain author and were selected independently and at random, but without overlapping. In the described experiments, the size of each training and experimental samples was 64 kB, and the experimental sample was divided into 16 fragments (slices). Hence, the size of any slice was 4 kB. (Experiments with different sizes of samples were also carried out.)

Denote the training sample as $X_{1},\ldots ,X_{17}$ and the slices as $Y_{j}^{i}$, where i=1,2,…,17 and j=1,2,…,16, that is, *i* corresponds to an author, *j* corresponds to a slice. Then we calculated the condition text compression ratios $\Psi (Y_{j}^{i}/X_{k})$, k=1,2,…,17, i=1,2,…,17 and j=1,2,…,16. (That is, we estimated the condition text compression ratio for any slice $Y_{j}^{i}$ and any training data $X_{k}$.) Then, we find the “closest writer” (CW) for any slice $Y_{j}^{i}$, that is calculated as follows

(1) $$CW\left(Y_{j}^{i}\right)=\min_{k=1,\ldots ,17}\Psi \left(Y_{j}^{i}/X_{k}\right)\;.$$

Results of those calculations are presented in Table 1. Let us, for example, look at the first arrow of the table. The number 15 means that $CW(Y_{j}^{1})$ was equal to 1 fifteen times, that is, the slices of the first writer (Pasternak) $Y_{j}^{1}$, j=1,…,16 were compressed better after their training sample $X_{1}$ ($CW(Y_{j}^{1})=1$ fifteen times). Besides, one time $CW(Y_{j}^{1})=13$. It means that one time the slice of the first writer (Pasternak) was better compressed after the training sample of the 13th author (Green). Note that for the “ideal” method of the author attribution, all diagonal elements should be 16, whereas all others should be 0.

The second stage: choosing parameters. As you can see, the results of the calculations performed can depend on such parameters as the size of the training sample, experimental and slice. Moreover, in some articles devoted to text classification, researchers suggest pre-removing numbers, punctuation and so-called “stop-words” from texts (that is, words that do not carry a semantic load: particles, conjunctions, etc.). Researchers do such text preparation in order to reduce the impact on the result of possible “text noise” [6]. Many researchers recommend formatting words according to the same register. Such text transformations can also be viewed as method parameters. In a sense, the data compressor used is a parameter of the method and must also be chosen experimentally.

We did many experiments with different parameters to create tables similar to Table 1 in order to choose the best parameters. To do this, we used the well-known statistical estimates of interdependencies [21] to find the table with the highest interdependency (corresponds to an ideal table where all non-zero values are on the main diagonal). More precisely, for Table 1 and all tables below, we tested the main hypothesis $H_{0}=\{$ all the data examined obey the same distribution} against the alternative hypothesis $H_{1}=$ negation $H_{0}$. The test for this problem is described, for example, in [21] (see also [14]).

In short, the test is as follows: there is a table

$$\begin{array}{cccc} a_{1\;1} & a_{1\;2} & \ldots & a_{1\;n}\\ a_{2\;1} & a_{2\;2} & \ldots & a_{1\;n}\\ \ldots \\ a_{m\;1} & a_{m\;2} & \ldots & a_{m\;n} \end{array}$$

and define

$$N=\sum_{i=1}^{n}\sum_{i=1}^{m}a_{i,j},p_{i,j}=a_{i,j}/N,p_{i.}=\sum_{j=1}^{n}p_{i,j},\;p_{.j}=\sum_{i=1}^{m}p_{i,j},$$

(2) $$x^{2}=\sum_{i=1}^{n}\sum_{i=1}^{m}\frac{{\left(a_{i,j}-Np_{i.}p_{.j}\right)}^{2}}{Np_{i.}p_{.j}}\;\;.$$

It is known that $x^{2}$ obeys the χ-square distribution $\chi_{\left(n-1)(m-1\right)}^{2}$ and the hypothesis $H_{0}$ must be rejected if $x^{2}$ is greater than the quantile $\chi_{(n-1)(m-1),\;\alpha }^{2}$, where α is the significance level [21].

Since the classification results can be affected by the preprocessing mentioned above, we investigated this problem. First of all, we removed unnecessary spaces, line breaks and unreadable characters from the texts—everything that the authors did not exactly add to their works themselves. In addition, we investigated all possible combinations of preprocessing: with punctuation/without punctuation, with stop-words/without stop-words, with capital letters/without capital letters. Then, we calculate the value $x^{2}$ in (2) for the tables similar Table 1 and obtain the following Table 2.

It can be seen from the table that for all types of preprocessing, the criterion values are very high, therefore the hypothesis $H_{0}$ about the uniformity of the distribution of values in the table is rejected with a significance level of 0.000001 in each case. Besides, it can be seen that the highest criterion value was achieved for texts from which only punctuation was removed. Therefore, this preprocessing was used in all other experiments.

Similar experiments were carried out to find the most suitable data compressor. Namely, we investigated archivers BZIP2, DEFLATE, LZMA, as we did with preprocessing methods, and LZMA was chosen based on these experiments.

We limited our experimentation with the choice of text compressors because our further research shows that LZMA is suitable. Namely, experiments make it possible to unambiguously establish the authorship (see the next part). On the other hand, additional research on data compressors can provide useful information. The point is that different data compressors can be developed for different languages. For example, there are compressors that are better in Slavic [22] than, say, in English.

The next investigated parameter was the sizes of the training sample and slices. We looked at training samples of 32, 64, 90, and 128 KB, and the slices ranged in size from 1 to 8 KB. The results show that the values of $x^{2}$ in (2) for training samples of 64, 90 and 128 kB are very close for all slice sizes. The effect of the slice size is negligible if it is larger than 2 KB.

So, based on all the experiments, the size of the training sample was determined as 64 KB, and the slice size was 4 KB (or 2 KB if the total size of the texts of a particular writer was limited). In general, the impact of all considered parameters on $x^{2}$ in (2) (i.e., type preprocessing, archivers, and training sample and slice size if they are larger than 64 kB and 2 kB, respectively) is very small. It is also important to note that the test (2) was applied to all tables in this article, and in all cases $H_{0}$ was rejected with a significance level of 0.000001.

It is also worth noting that this stage should be used to solve any specific attribution problem, and the results may depend on the literary works in question.

The last step: the author attribution of literary texts. Let us return to analysis of the works “The Twelve Chairs” and “The Golden Calf”. As noted earlier, the hypothesis has recently become widespread that the works “The Twelve Chairs” and “The Golden Calf” were written by M. Bulgakov, and not I. Ilf and E. Petrov. Now we will test this hypothesis using the method described above. First, let us test our method by applying it to literary works with undoubted authorship. Table 2 shows the results of the application of the investigated method. There, a subset of all texts by I. Ilf and E. Petrov with a total volume of 128 kB, and a subset of all works by M. Bulgakov, from which “Heart of a Dog” was excluded, was used as a training sample. As a slice, we used 2 kB fragments of “Heart of a Dog”. The total size of the test sample is 128 kB (that is, there were 64 slices in the test sample).

From the table, one can make an unambiguous conclusion about the authorship of the work “Heart of a Dog”, which in this case indicates the quality of the method.

Next, we conducted a study of the differentiation of the styles of the studied authors. Without changing the training sample, we took 128 kB of arbitrary texts from each of the authors as a test sample. Table 3 shows the results of this experiment.

It can be seen from the table that the authors have unique, distinguishable styles. (Furthermore, of course, this is statistically confirmed.) This means that the authorship of the works written by them should also be unambiguously determined within the framework of the experiment. Finally, let us find out the authorship of the works “The Twelve Chairs” and “The Golden Calf”. Table 4 shows the results of the experiment.

In this case, the hypothesis of data homogeneity is rejected with a significance level of 0.00001. As can be seen from Table 5, the results obtained by the described method indicate that the authorship of “The Twelve Chairs” and “The Golden Calf” belongs to I. Ilf and E.Petrov. However, in contrast to the experiment with the work “Heart of a Dog”, some of the blocks were still assigned to M. Bulgakov. Apparently, the works under study are indeed insignificantly similar to the works of M. Bulgakov, which can be explained by the friendship of the writers and the possible influence of Bulgakov as a recognized literary leader.

Brief formal description of the literary text attribution method. The proposed method was described along with a solution to a specific attribution problem. In this short part, we will summarize the method description to present it as it is.

(**i**) *Compile a collection of works of writers who lived simultaneously with considered ones.*

(**ii**) *Prepare the texts of the selected authors as follows: divide each of them into two parts (training and experimental).* Both parts should be composed of different works by a specific author and should be selected independently and at random, but without duplication.

The initial size of each training sample and experimental samples is 64 kB, and the experimental sample is divided into 16 fragments (slices). Therefore, the initial size of any slice was 4KB. For any training part $X_{i}$ and slice $Y_{m}^{n}$, calculate $\Psi (Y_{m}^{n}/X_{i})$, where i,n=1,…,N, m=1,…,M and *N* are the number of authors, and *M* is the number of text slices written by one writer. ($\Psi (Y_{m}^{n}/X_{i})$ is defined in Preliminary stage.) Then, calculate $CW\left(Y_{j}^{i}\right)=\min_{k=1,\ldots ,17}\Psi \left(Y_{j}^{i}/X_{k}\right)\;$ and $x^{2}$ (see (1) and (2).

(**iii**) *Optimization parameters step.* Repeat (**ii**) with different sizes of training and experimental parts and slices, as well as with different data compressors and types of preprocessing and find the parameters for which $x^{2}$ is the maximum.

(**iv**) *The author attribution step.* According to the selected parameters, carry out the attribution of the studied literary works in such a way that the training part is taken from the investigated works, and the slices—from the works of both authors. Step (**ii**) is then performed with this data and a decision can be made based on the results. The significance level is determined based on the calculated $x^{2}$, see (2).

## 4. Analysis of Literary Works “Three Countries of the World” and “Dead Lake” by A. Nekrasov and A. Panaeva

The question of the authorship of the novels “Three countries of the world” and “Dead Lake”, published in the journal “Sovremennik”, respectively, in 1848 and 1851, has long been of little interest to researchers. For the first time, this issue became acutely relevant during the preparation of both novels for the publication of the “Complete Works and Letters of N. Nekrasov in 15 volumes” in 1981 [23]. In “Memoirs” A. Panaeva reports that the authors of the novel “Three Countries of the World” are both writers: Nekrasov, Panaeva; but the novel “Dead Lake” was written by her with insignificant participation of Nekrasov.

We know from history that the decision to write novels was made due to an acute shortage of literary works for publication in the magazine “Sovremennik”. Since 1848, the official editor of the magazine was Ivan Panaev, and N. Nekrasov was its co-editor. That is why they decided to quickly write two novels for publication in “Sovremennik”. A. Panaeva offered her help in writing novels. History is silent about the contribution of each of the three writers to the creation of the novels. However, I. Panaev took part in the creation of novels absolutely for sure. This fact is known for certain and does not cause doubts among literary critics. Therefore, we also included I. Panaev as a possible author in the our investigation.

Based on the philological analysis of the text, literary researchers, contrary to Panaeva’s testimony, attribute a substantial part of the text to Nekrasov in both novels (indicating specific chapters). Furthermore, since literary researches did not come to an accurate conclusion regarding the author’s attribution of the text, researchers began to use mathematical methods to solve this problem.

In this part, we will apply the above method to this problem. First of all, we selected almost all the writers who wrote at about the same time as Nekrasov and Panaeva, then we determined a more suitable data compressor and the sizes of training parts and slices. It turned out that the maximum value of $x^{2}$ in (2) was obtained with a training sample size of 128 kB, the number of slices is 16, and the size of each is 8 kB. The results are shown in Table 6:

Then, we applied the proposed method with 128-kB size and 8-kB slices for the authorship of the novels “Three Countries of the World” and “Dead Lake”. The obtained results are presented below.

Table 7 shows that the authors of the novel “Three Countries of the World” were three writers: A. Panaeva, N. Nekrasov, and I. Panaev. The discovered fact does not contradict the statements of literary critics. We have proved that Part 5 andPpart 8 were written by N. Nekrasov, and the conclusion is attributed to I. Panaev. All other parts, including “Prologue”, were written by A. Panaeva. This is most of the text. She can be considered the main author of the text, and not N. Nekrasov, as was previously assumed in literary criticism.

Table 8 shows that the authorship of I. Panaev is absolutely excluded. He did not take part in the writing of the novel “Dead Lake”. Parts 6, 11, and 12 are attributed to N. Nekrasov. Perhaps he dictated these parts of the novel, and she wrote it down, since they lived together and worked together. The entire main text of the novel “Dead Lake” was written by A. Panaeva. She is the true author of this novel.

## 5. Conclusions

This paper proposes a method of attribution of literary texts, which is based on the information-theoretic solution of the “three-sample problem” [14], as well as classical statistical tests of homogeneity. However, the proposed method is a significant extension of this test. We also note that the proposed method has some limitations associated with the possible lack of the required number of literary texts. (For example, for ancient literary works).

It is worth noting that in our examples, we estimated the parameters for two different attribution tasks and, interestingly, it turned out that some parameters are different. Namely, the slice sizes were 4 KB and 8 KB, respectively. It should be noted that the works in question were written in different centuries (XIX and XX, respectively) and, perhaps, the difference in parameters can be explained by possible changes in the literary style and even language.

In general, the performed experiments also show that the data compression method can be used for identifying authorship together with traditional strategies in literary studies.

## Figures and Tables

**Table 1:** Results of the experiments. 1—Pasternak, 2—Nabokov, 3—Paustovsky, 4—Veresayev, 5—Zamyatin, 6—Bunin, 7—Gorky, 8—Kuprin, 9—Platonov, 10—Mandelstam, 11—Olesha, 12—Katayev, 13—Green, 14—Zhitkov, 15—Ehrenburg, 16—Tynyanov, 17—Gaidar.

	1	2	3	4	5	6	7	8	9	10	11	12	13	14	15	16	17
1	15	0	0	0	0	0	0	0	0	0	0	0	1	0	0	0	0
2	0	12	0	0	0	0	0	0	0	0	0	0	4	0	0	0	0
3	0	0	15	0	0	0	0	0	0	0	0	0	1	0	0	0	0
4	0	0	0	16	0	0	0	0	0	0	0	0	0	0	0	0	0
5	0	0	0	0	14	0	0	0	0	1	0	1	0	0	0	0	0
6	0	1	0	0	0	12	0	0	0	1	0	0	2	0	0	0	0
7	0	0	0	0	0	0	16	0	0	0	0	0	0	0	0	0	0
8	0	0	0	0	0	0	1	10	0	0	0	1	4	0	0	0	0
9	0	0	0	0	0	0	0	0	16	0	0	0	0	0	0	0	0
10	0	0	0	0	0	0	0	0	0	16	0	0	0	0	0	0	0
11	0	0	0	0	0	0	0	0	0	0	16	0	0	0	0	0	0
12	0	0	0	0	0	0	0	0	0	0	0	16	0	0	0	0	0
13	0	0	0	0	0	0	0	0	0	0	0	0	16	0	0	0	0
14	0	0	0	0	0	0	0	0	0	0	0	0	0	16	0	0	0
15	0	0	0	0	0	0	0	1	0	2	0	0	1	0	12	0	0
16	0	0	0	0	0	0	0	0	0	0	0	0	0	0	0	16	0
17	0	0	0	0	0	0	0	0	0	0	0	0	1	0	0	0	15

**Table 2:** Results of the experiments with preprocessing.

Type of Text Preprocessing	$x^{2}$
Without punctuation, without stop-words, without capital letters	3608.9
Without punctuation, with stop-words, without capital letters	3788.9
Without punctuation, without stop-words, with capital letters	4061.2
Without punctuation, with stop-words, with capital letters	4146.7
With punctuation, without stop-words, without capital letters	3935.2
With punctuation, without stop-words, with capital letters	3903.7
With punctuation, with stop-words, without capital letters	3536.9
With punctuation, with stop-words, with capital letters	4133.2

**Table 3:** Attribution of the work “Heart of a Dog”.

Authors	M. Bulgakov	I. Ilf and E.Petrov
«Heart of a Dog»	64	0

**Table 4:** Cross-check of the tested texts.

Authors	M. Bulgakov	I. Ilf and E.Petrov
M. Bulgakov	63	1
I. Ilf and E.Petrov	0	64

**Table 5:** Attribution of literary works “The Twelve Chairs” and “The Golden Calf”.

Authors	M. Bulgakov	I. Ilf and E.Petrov
“The Twelve Chairs”	7	57
“The Golden Calf”	5	59

**Table 6:** Nekrasov—1, Panaeva—2, Turgenev—3, Goncharov—4, Gogol—5, Dahl—6, Dostoevsky—7, Panaev—8, S.-Shchedrin—9, Tolstoy—10, Tolbin—11, Druzhinin—12.

	1	2	3	4	5	6	7	8	9	10	11	12
1	12	2	1	0	0	1	0	0	0	0	0	0
2	1	10	5	0	0	0	0	0	0	0	0	0
3	0	0	16	0	0	0	0	0	0	0	0	0
4	0	0	2	12	0	0	0	1	0	0	0	1
5	0	0	0	0	15	0	0	0	0	1	0	1
6	1	0	0	1	1	13	0	0	0	1	0	0
7	0	0	1	0	0	0	11	1	2	0	0	1
8	0	0	1	0	0	0	1	14	0	0	1	0
9	0	0	1	4	0	0	0	2	12	0	0	0
10	0	2	0	0	0	0	0	0	0	14	0	0
11	0	0	0	0	0	0	0	4	0	0	11	1
12	1	0	0	0	0	0	2	0	0	0	0	13

**Table 7:** Results of the study of attribution of parts of the novel “Three Countries of the World” (16 slices of 8 kb). 1—Prologue, 2—Part 1, 3—Part 2, 4—Part 3, 5—Part 4, 6—Part 5, 7—Part 6, 8—Part 7, 9—Part 8, 10—Conclusion.

	1	2	3	4	5	6	7	8	9	10
Nekrasov	4	2	2	6	4	10	2	2	10	2
Panaeva	12	13	14	10	12	6	14	14	6	2
Panaev	0	1	0	0	0	0	0	0	0	14

**Table 8:** Results of the study of attribution of the novel “Dead Lake” (16 slices of 8 kb). Here N.—Nekrasov, Pa—Panaeva, P—Panaev; 1—Part 1, 2—Part 2, 3—Part 3, 4—Part 4, 5—Part 5, 6—Part 6, 7—Part 7, 8—Part 8, 9—Part 9, 10—Part 10, 11—Part 11, 12—Part 12, 13—Part 13, 14—Part 14, 15—Part 15, 16—Epilogue.

	1	2	3	4	5	6	7	8	9	10	11	12	13	14	15	16
N.	2	3	4	1	2	13	0	0	2	6	10	12	0	3	2	3
Pa.	14	13	12	15	14	3	16	16	14	10	6	4	16	13	14	13
P.	0	0	0	0	0	0	0	0	0	0	0	0	0	0	0	0

## References

[1] Lowe D., Matthews R. (1995). Shakespeare vs. Fletcher: A Stylometric Analysis by Radial Basis Functions. Comput. Humanit..

[2] Ryabko B., Astola J., Malyutov M. (2016). Compression-Based Methods of Statistical Analysis and Prediction of Time Series.

[3] Khmelev D.V. (2003). Classification and Mark Up of Texts Using Data Compression Methods. All about Data, Image and Video Compression. https://www.compression.ru/download/articles/classif/intro.html.

[4] Oliveira J.W., Justino E., Oliveira L. (2013). Comparing compression models for authorship attribution. Forensic Sci. Int..

[5] Kukushkina V., Polikarpov D.V., Khmelev D.V. (2001). Text authorship attribution using letters and grammatical information. Probl. Inf. Transm..

[6] Gorshkov S., Nered M., Ilyushin E., Namiot D. (2019). Using Machine Learning Methods to Establish Program Authorship. International Journal of Open Information Technologies. No1. https://cyberleninka.ru/article/n/using-machine-learning-methodsto-establish-program-authorship.

[7] Marusenko M.A., Bessonov B.A., Bogdanova L.M., Myasoedova N.E. (2001). Search for the Lost Author, Attribution Etudes.

[8] Shannon C.E. (1948). A mathematical theory of communication. Bell Syst. Tech. J..

[9] Teahan W.J., Wen Y.Y., McNabb R., Witten I.H. (2000). Using compression models to segment Chinese text. Comput. Linguist..

[10] Teahan W.J., Harper D.J. (2003). Using compression- based language models for text categorization. Language Modeling for Information Retrieval.

[11] Cilibrasi R., Vitanyi P. (2005). Clustering by compression. IEEE Trans. Inf. Theory.

[12] Cilibrasi R., Vitanyi P., De Wolf R. (2004). Algorithmic clus- tering of music based on string compression. Comput. Music.

[13] Ryabko B.Y., Guskov A.E., Selivanova I.V. (2017). Information-Theoretic Method for Classification of Texts. Probl. Inf. Transm..

[14] Ryabko B.Y., Guskov A.E., Selivanova I.V. Using data-compressors for statistical analysis of problems on homogeneity testing and classification. Proceedings of the 2017 IEEE International Symposium on Information Theory (ISIT).

[15] Amlinsky I. (2013). 12 Chairs from Mikhail Bulgakov.

[16] Kozarovetskiy V.A., Moscow Baranki and Odessa Bubliki (2013). Who Wrote “12 Chairs” Literary Russia, No. 41. http://old.litrossia.ru/2013/41/08347.html.

[17] Khmelnitsky D.S. (2014). In Defense of Ilf and Petrov Seven Arts. v. 52. http://7iskusstv.com/2014/Nomer5/Chmelnicky1.php.

[18] Freidgeym L.I. (2014). Ilf and Petrov or Bulgakov … Round Table (Virtual Version) Seven Arts, v. 47. http://7iskusstv.com/2013/Nomer11/Frejdgejm1.php.

[19] Bessonov B.L. (1978). On the Authorship of the Novel “Three Countries of the World”.

[20] Panaeva A.Y. Memories. http://az.lib.ru/p/panaewa.

[21] Kendall M., Stjuart A. (1961). The Advanced Theory of Statistics.

[22] Lansky J., Zemlicka M. Text compression: Syllables. Proceedings of the Dateso 2005 Annual International Workshop on DAtabases, TExts, Specifications and Objects.

[23] Nekrasov N.A. (1981). The Complete Collection of Works and Letters: In 15 Vols. Artistic Works.

